# Molecular pathways in mitochondrial disorders due to a defective mitochondrial protein synthesis

**DOI:** 10.3389/fcell.2024.1410245

**Published:** 2024-05-24

**Authors:** Álvaro Antolínez-Fernández, Paula Esteban-Ramos, Miguel Ángel Fernández-Moreno, Paula Clemente

**Affiliations:** ^1^ Instituto de Investigaciones Biomédicas Sols-Morreale (IIBM), Universidad Autónoma de Madrid-Consejo Superior de Investigaciones Científicas, Madrid, Spain; ^2^ Departamento de Bioquímica, Universidad Autónoma de Madrid, Madrid, Spain

**Keywords:** mitochondria, translation, mitoribosome, OxPhos, mitochondrial disorders

## Abstract

Mitochondria play a central role in cellular metabolism producing the necessary ATP through oxidative phosphorylation. As a remnant of their prokaryotic past, mitochondria contain their own genome, which encodes 13 subunits of the oxidative phosphorylation system, as well as the tRNAs and rRNAs necessary for their translation in the organelle. Mitochondrial protein synthesis depends on the import of a vast array of nuclear-encoded proteins including the mitochondrial ribosome protein components, translation factors, aminoacyl-tRNA synthetases or assembly factors among others. Cryo-EM studies have improved our understanding of the composition of the mitochondrial ribosome and the factors required for mitochondrial protein synthesis and the advances in next-generation sequencing techniques have allowed for the identification of a growing number of genes involved in mitochondrial pathologies with a defective translation. These disorders are often multisystemic, affecting those tissues with a higher energy demand, and often present with neurodegenerative phenotypes. In this article, we review the known proteins required for mitochondrial translation, the disorders that derive from a defective mitochondrial protein synthesis and the animal models that have been established for their study.

## 1 Introduction

Mitochondria are eukaryotic organelles that play a central role in cellular metabolism, participating in key cellular processes, from ATP synthesis through oxidative phosphorylation (OXPHOS), the biosynthesis of nucleotides and amino acids or lipid metabolism to reactive oxygen species generation and apoptosis (Spinelli and Haigis, 2018).

Mitochondria originated from α-proteobacteria that were incorporated into a eukaryotic cell and have evolved as endosymbionts over billions of years. As a remnant of their prokaryotic past, mitochondria maintain a small genome, the mitochondrial DNA (mtDNA). Human mtDNA is a small circular double-stranded DNA molecule that encodes 13 essential subunits of the OXPHOS complexes. Mitochondria contain their own translation machinery, which is solely dedicated to the translation of the 13 mtDNA encoded proteins. mtDNA encodes two rRNAs, components of the mitochondrial ribosome, and 22 tRNAs that are required for mitochondrial translation. The rest of the ∼1100 proteins that compose the mitochondrial proteome, including the ∼65 remaining OXPHOS subunits, and the proteins required for a correct expression of mtDNA, are encoded on the nuclear genome, are translated in the cytosolic ribosomes and imported into mitochondria (Vafai and Mootha, 2012). This separation of the OXPHOS-encoding genes and the genes responsible for their expression in two different cellular compartments, requires that the cell coordinates their gene expression machineries to adapt the biogenesis of the OXPHOS complexes to the cells’ energy demand (Couvillion et al., 2016).

Transcription of the mitochondrial genome produces polycistronic transcripts that cover almost the entire length of the mtDNA molecule. Over 40 years ago, work by the group of Giuseppe Attardi showed that most mitochondrial protein-coding genes and rRNAs were immediately flanked by tRNA coding sequences, without any intergenic space, which led them to propose that tRNAs act as punctuation marks for the processing of these transcripts (Ojala et al., 1980; 1981; Montoya et al., 1981). Since then, it has been shown that individual RNA molecules are released from this polycistronic transcripts by the concerted action of two endonucleases, RNase P and RNase Z (also known as ELAC2), that recognize the tRNA structure within these transcripts (Holzmann et al., 2008; Brzezniak et al., 2011). These enzymes cleave on the 5′ and 3’ end of the tRNAs respectively, releasing the mRNAs and rRNAs (Rackham et al., 2016). There are however certain gene junctions in the polycistronic mitochondrial transcripts that do not contain a tRNA and the details and protein factors involved in their processing are now starting to emerge (Ohkubo et al., 2021; Clemente et al., 2022). The newly processed RNAs need to be further modified for proper maturation and rRNAs are assembled together with the ribosome protein components to form the mature mitoribosome.

The correct expression of the OXPHOS genes encoded in the mitochondrial genome, therefore, depends on a correct mtDNA maintenance and transcription, RNA maturation, mRNA stability, ribosomal biogenesis, and translation. Given its importance for the biogenesis of the OXPHOS complexes, an impairment in any of the processes involved in mitochondrial gene expression results in pathological situations, including devastating disorders. Mitochondrial disorders caused by a defective mitochondrial protein synthesis can arise from mutations in the mitochondrially encoded tRNAs and rRNAs, but also from mutations in nuclear genes encoding mitorribosomal proteins (MRPs) and ribosome assembly factors, translation factors, and mitochondrial aminoacyl tRNA synthetases, among others. These mutations generally result in a combined defect of the OXPHOS enzymes, and clinical manifestations such as Leigh syndrome, sensorineural hearing loss, encephalomyopathy, and hypertrophic cardiomyopathy. In this review we will focus on the defects that impair mitochondrial translation leading to mitochondrial disorders and the animal models generated for their study.

## 2 Structure and biogenesis of the mitochondrial ribosome

Protein synthesis activity within mitochondria was identified in the late 1950s (McLean et al., 1958) and in 1967, mitochondrial ribosomes (mitoribosomes) were isolated from the fungus *Neurospora crassa* (Küntzel and Noll, 1967) and rat liver mitochondria (O’Brien and Kalf, 1967a; O’Brien and Kalf, 1967b). But it has not been until the 2010s, that the advances in cryo-electron microscopy (Cryo-EM) allowed the determination of the structure of the yeast (Desai et al., 2017), porcine (Greber et al., 2015) and human mitoribosomes (Amunts et al., 2015).

Mitochondrial ribosomes have diverged substantially from their bacterial ancestors and their structure differs from known bacterial and eukaryotic cytosolic ribosomes. Mitoribosomes have a higher protein content that their bacterial counterparts, ∼ 1:2 rRNA:protein ratio for the mammalian mitochondrial ribosome. The mammalian 55S mitoribosome is composed of two subunits, a small 28S subunit (mt-SSU) which contains 12S rRNA and 30 nuclear-encoded mitochondrial ribosomal proteins (MRPs), and a large 39S subunit (mt-LSU), which includes 16S rRNA and 52 nuclear-encoded MRPs and either tRNA^Val^ (in humans or rat) or tRNA^Phe^ (in porcine or bovine ribosomes), which has been recruited to the ribosome in the site of bacterial 5S (Rorbach et al., 2016; Kummer and Ban, 2021). Mitoribosomal proteins are primarily found on the periphery of the ribosome, surrounding the catalytic core.

Mitoribosome biogenesis takes place in the mitochondrial matrix, in the so-called RNA granules, near the mtDNA nucleoids. This process requires a set of auxiliary factors that include GTPases, helicases, kinases and modifying enzymes, which catalyze the assembly of the mitoribosomal subunits with the mitochondrially encoded rRNAs. Cryo-EM studies have been instrumental in deciphering the process of mitoribosome assembly, revealing the conformational changes in rRNA and the hierarchical incorporation of MRPs that are required to form the mature mitoribosome. It also allowed to visualize the binding of the assembly factors during the different steps of mitoribosome formation, giving insights into their molecular function.

**FIGURE 1:**
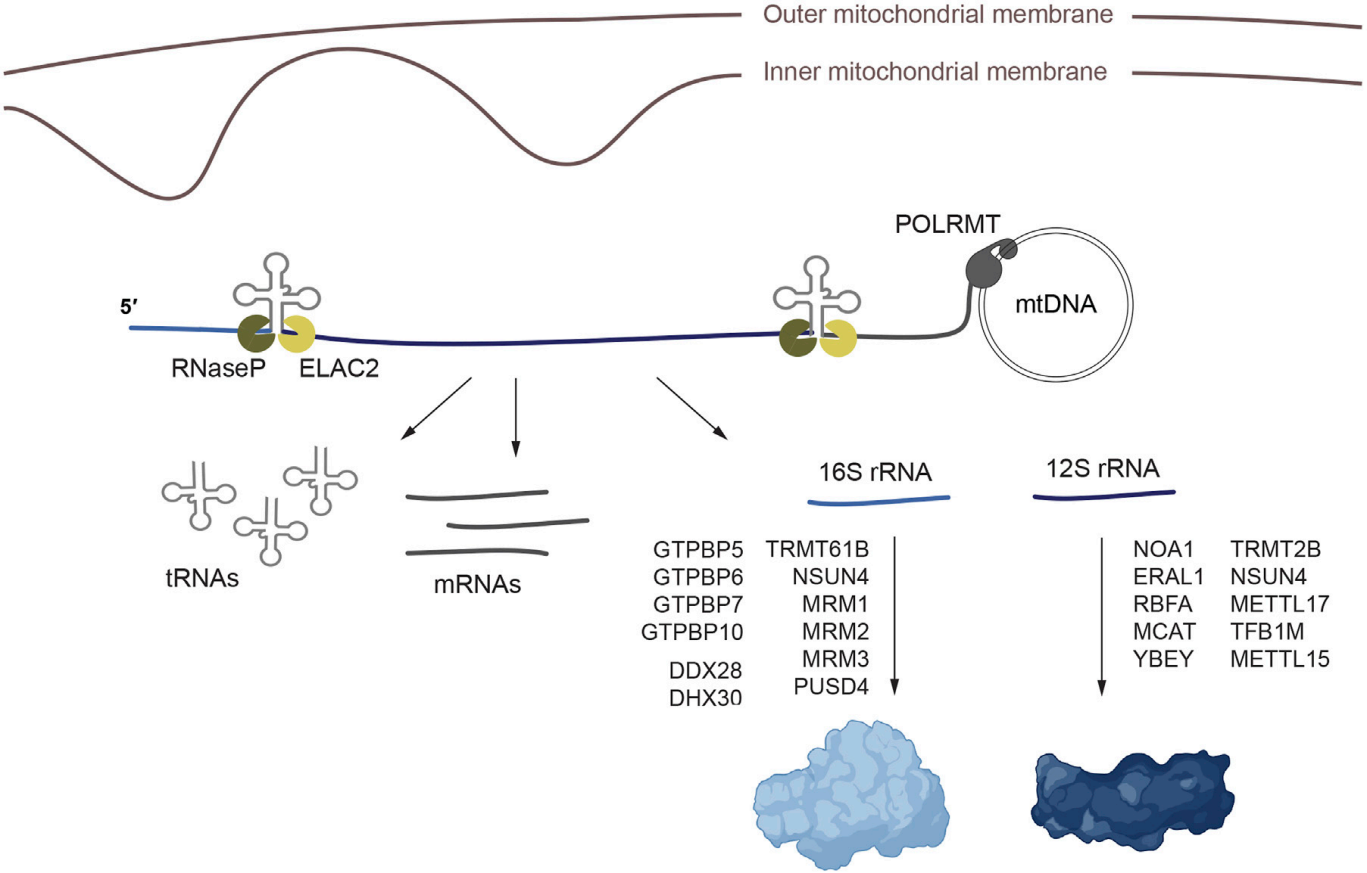
Processing and maturation of the mitochondrial rRNAs and assembly of the mitorribosome. Mitochondrial transcription generates polycistronic precursors that are processed to release the individual rRNA, tRNA and mRNA molecules. Once the rRNAs are processed, mt-SSU and mt-LSU assembly proceeds aided by methyltransferases, RNA helicases, GTPases and additional assembly factors. The ribosome figure was created with BioRender.

The assembly of mt-SSU requires the participation of methyltransferases (TRMT2B, NSUN4, METTL17, TFB1M and METTL15), GTPases (NOA1/MTG3, ERAL1), the rRNA chaperone RBFA, the endoribonuclease YbeY and malonyl-CoA-acyl carrier protein transacylase (MCAT) (Figure 1). The GTPases NOA and ERAL1 promote the folding of the 12S rRNA in the initial steps of mt-SSU assembly, to form the ribosome platform and the decoding center (region of the SSU where codon-anticodon pairing takes place) (Dennerlein et al., 2010; Uchiumi et al., 2010; Kolanczyk et al., 2011; He et al., 2012; Summer et al., 2020; D’Souza et al., 2021; Harper et al., 2023). The initial maturation of the mt-SSU also requires METTL17 and MCAT, which coordinate the maturation of the rRNA (Shi et al., 2019; Harper et al., 2023; Ast et al., 2024). RBFA binds the immature mt-SSU and facilitates the incorporation of the methyltransferase TFB1M, which methylates two highly conserved adenines, A936 and A937, in 12S rRNA, and METTL15, which methylates 12S rRNA at position C839 (Seidel-Rogol et al., 2003; Haute et al., 2019; Itoh et al., 2022; Harper et al., 2023). Biochemical studies have revealed that the assembly of mt-SSU requires additional factors, although the precise assembly intermediate with which they interact remains to be established. This is the case of the endoribonuclease YbeY, which incorporates the uS11 subunit into mt-SSU (Summer et al., 2020; D’Souza et al., 2021) or the methyltransferases TRMT2B (Laptev et al., 2020; Powell and Minczuk, 2020) and NSUN4 (Cámara et al., 2011; Metodiev et al., 2014) which methylate positions U429 and C841 respectively.

The assembly of mt-LSU, on the other hand, requires the GTPases GTPBP5, GTPBP6, GTPBP7 and GTPBP10, the methyltransferases TRMT61B, NSUN4, MRM1, MRM2 and MRM3, the pseudourydilase RPUSD4, the RNA helicase DDX28, the mitochondrial transcription termination factor family protein mTERF4, and a module composed of the mitochondrial assembly of ribosomal large subunit 1 (MALSU1), the leucine-tyrosine-arginine motif family protein L0R8F8 and the mitochondrial acyl carrier protein mt-ACP (Figure 1). GTPases GTPBP5, GTPBP6, GTPBP7 and GTPBP10 participate in the late stages of the assembly of mt-LSU (Kim and Barrientos, 2018; Lavdovskaia et al., 2018; Lavdovskaia et al., 2020; Maiti et al., 2018; Maiti et al., 2020; Cipullo et al., 2020). The methyltransferase TRMT61B methylates position methylation of A947 in 16S rRNA (Bar-Yaacov et al., 2016). Methyltransferases MRM1, MRM2 and MRM3 catalyze the 2′-O-ribose methylation of positions G1145, U1369 and G1370 which are necessary to stabilize or induce conformational changes in 16S rRNA (Lee and Bogenhagen, 2014; Rorbach et al., 2014). MTERF4 and NSUN4 form a complex that methylates 12S rRNA and that participates in mtLSU assembly (Cámara et al., 2011; Spåhr et al., 2012; Metodiev et al., 2014). The RNA helicase DDX28 interacts with 16 rRNA, stabilizing the central protuberance in an immature conformation (Antonicka and Shoubridge, 2015; Tu and Barrientos, 2015; Cheng et al., 2021). The pseudourydilation modification introduced by RPUSD4 is essential for 16S rRNA stability and assembly into mt-LSU (Arroyo et al., 2016; Antonicka et al., 2017; Zaganelli et al., 2017). MALSU1, L08R8F8 and mt-ACP form a module that binds mt-LSU preventing its premature assembly with mt-SSU (Brown et al., 2017).

In addition to these auxiliary factors, several studies have identified additional proteins involved in mitochondrial 16 rRNA modification or the assembly of the mt-LSU, although their precise function or the step of the assembly in which they participate remains unknown. This is the case of the RNA helicase DHX30 (Antonicka and Shoubridge, 2015), MPV17L2 (Rosa et al., 2014) and RCC1L/WBSCR16, NGRN, FASTKD, RPUSD3 which form a module with RPUSD4 (Antonicka and Shoubridge, 2015; Popow et al., 2015; Arroyo et al., 2016; Antonicka et al., 2017; Reyes et al., 2020).

Mitorribosome structure and the mitoribosomal assembly have been extensively reviewed in (Ferrari et al., 2021; Maiti et al., 2021; Khawaja et al., 2023).

## 3 Mitochondrial translation

Mitochondrial translation is a multistep process that involves the canonical steps of initiation, elongation, termination and recycling of the ribosome, driven by a set of translation factors that interact with the ribosome in each of these steps of the process.

### 3.1 Translation initiation

Translation initiation comprises the necessary steps for the recognition of the start codon of the mRNA and the addition of the first aminoacyl-tRNA. The start codon must be placed at the peptidyl site (P-site) of the ribosome in order to stablish the correct reading frame of the transcripts.

**FIGURE 2:**
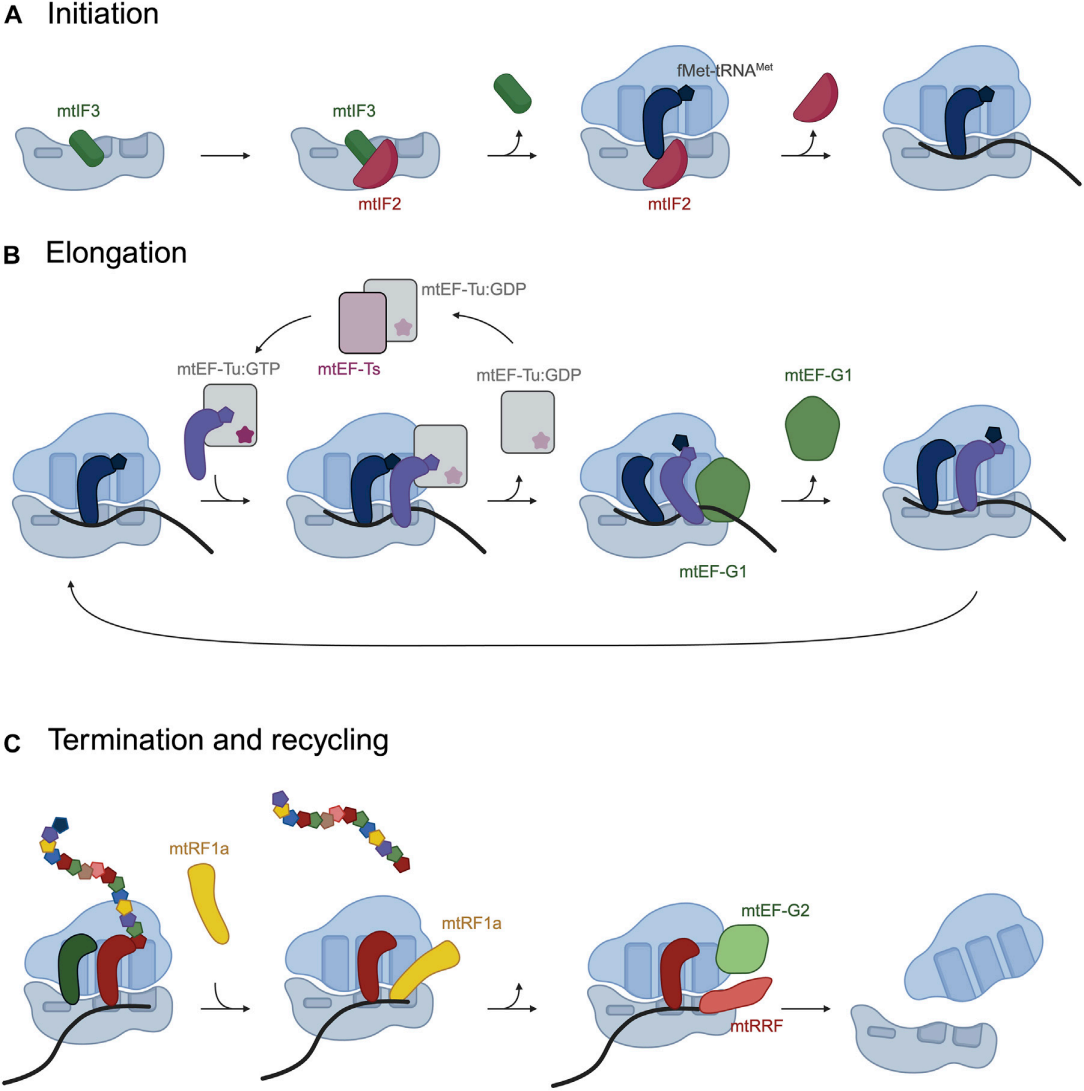
Schematic representation of the phases in mitochondrial translation and the translation factors that assist in the process. **(A)** During translation initiation, mitochondrial initiation factors mtIF3 and mtIF2 bind the ribosome and fMet-tRNA^fMet^ is recruited to the P-site. **(B)** Translation elongation consists on cycles in which mtEF-Tu:GFP delivers an aminoacyl-tRNA to the A-site of the ribosome. mtEF-G1 catalyzes the translocation of the ribosome and contacts the tRNA-mRNA base pairing to maintain the reading frame. **(C)** mtRF1a recognizes UAA or UAG at the A-site and terminates translation. mtEF-G2 and mtRRF split the mitoribosomal subunits. Figure 2 was created with BioRender.

In bacteria, three translation initiation factors, IF1, IF2 and IF3, bind the small ribosomal subunit and recruit a devoted initiator aminoacyl tRNA, fMet-tRNA^fMet^. In mitochondria, in contrast, there are just two initiation factors, mtIF2 and mtIF3, which have acquired specific insertions and extensions. Cryo-EM studies have described a first pre-initiation complex in which mtIF3 binds the mt-SSU preventing the binding of the initiator fMet-tRNA^Met^ and suggest it dissociates before the binding of the mRNA and fMet-tRNA^Met^ (Figure 2A) (Khawaja et al., 2020; Itoh et al., 2022). mtIF2 is subsequently recruited to the mt-SSU and prevents premature binding of the aminoacyl-tRNAs to the aminoacyl site (A site) of the mitoribosome, replacing the function of bacterial IF1, through a 37 amino acid insertion (Gaur et al., 2008; Yassin et al., 2011; Kummer et al., 2018). mtIF2 facilitates binding of fMet-tRNA^Met^ to the P-site by specific interactions with the formyl modification on the methionine. In human mitochondria three codons AUG, AUU and AUA are recognized as start codons. In mammalian mitochondria, the three initiator codons are recognized by a single tRNA^Met^ in which the wobble base of the anticodon has been modified to formylcytosine (Haute et al., 2016).

Together with the absence of IF1 and the presence of three initiator codons, there are further features that differentiate mitochondrial and bacterial translation initiation. Bacterial transcripts are loaded on the ribosome through an interaction of the 16S rRNA with a Shine-Dalgarno sequence located upstream of the mRNA initiator codon. Mitochondrial transcripts, however, are leaderless, carrying no or very short 5′ untranslated regions (5′UTRs) and how the mitochondrial mRNAs are loaded on the ribosome remains under investigation. Cryo-EM studies have shown that the mRNA is stably bound once mtIF3 has left the mt-SSU and the monosome is fully assembled (Kummer et al., 2018; Khawaja et al., 2020). Supporting this data, recent *in vitro* studies have shown that leaderless mitochondrial mRNAs preferentially bind the fully assembled 55S ribosome, rather than the small subunit (Remes et al., 2023). The mitoribosomal subunit mS39, a pentatricopeptide repeat (PPR) protein, crowns the mRNA entrance and has been proposed to promote the binding of the mRNA by interacting with a uridine stretch in the coding sequence of the transcript (Kummer et al., 2018).

### 3.2 Translation elongation

Once the monosome is formed and the initiator fMet-tRNA^Met^ has recognized the translational start codon, the ribosome moves along the mRNA adding amino acids to the growing polypeptide chain. Elongation takes place by subsequent cycles of codon recognition and aminoacyl-tRNA binding, peptide bond formation and translocation along the mRNA.

Elongation requires the participation of the mitochondrial elongation factor Tu (mtEF-Tu), with the assistance of the mitochondrial elongation factor Ts (mtEF-Ts) and the mitochondrial elongation factor G1 (mtEF-G1). During elongation, mtEF-Tu binds aminoacylated tRNAs and delivers them to the A-site of the ribosome (Figure 2B). Once the correct codon-anticodon pairing occurs, mtEF-Tu hydrolyzes GTP and leaves the mitoribosome. mtEF-Tu requires the assistance of a guanine exchange factor, mtEF-Ts, which catalyzes the exchange of GDP for GTP on mtEF-Tu preparing it for subsequent rounds of aminoacyl-tRNA delivery (Figure 2B) (Schwartzbach and Spremulli, 1989; Woriax et al., 1997). After peptide bond formation, the peptidyl-tRNA needs to be repositioned from the A- to the P-site of the mitoribosome, together with the mRNA. The translocation is catalyzed by mtEF-G1, with the hydrolysis of GTP (Bhargava et al., 2004; Kummer and Ban, 2020). The mitoribosome translocates the peptydil-tRNA and the mRNA by a rotational motion of the mt-SSU. During translocation, mtEF-G1 contacts the tRNA-mRNA base pairing to maintain the reading frame during translocation. This movement of the ribosome places the next codon of the mRNA in the A-site, ready to accept the next aminoacyl-tRNA.

A fourth elongation factor, mtEF4/GUF1, has been described to promote protein synthesis under stress conditions, improving the fidelity of the translation process (Bauerschmitt et al., 2008).

### 3.3 Translation termination and ribosome recycling

The elongation of the polypeptide proceeds until a STOP codon is placed in the A-site of the ribosome. In the universal genetic code UAA, UAG and UGA serve as stop codons. In human mitochondria, UAA and UAG maintain their conventional role as stop codons. UGA, however, encodes tryptophan and two additional codons AGA and AGG serve as translational stop codons.

Four putative translation termination factors have been identified in mitochondria: mtRF1a, mtRF1, ICT1 and C12ORF65, based on their homology to bacterial release factors. mtRF1a recognizes and terminates translation at codons UAA and UAG (Soleimanpour-Lichaei et al., 2007), but it does not recognize the “non-canonical” stop codons AGA and AGG in *COX1* and *ND6* transcripts respectively (Figure 2C). It was proposed that termination at *COX1* and *ND6* occurs through frameshifting of the mitoribosome due to the absence of tRNAs that recognize AGA and AGG codons in mitochondria (Temperley et al., 2010). In both cases, a −1 frameshift would place a conventional UAG STOP codon at the A site of the ribosome, which would mean translation of all mitochondrially encoded peptides terminates at either UAA or UAG. In several vertebrate species, however, AGA and AGG are not preceded by U, questioning whether the −1 frameshift occurs. Two recent publications have demonstrated thar mtRF1 is the release factor responsible for termination at the AGA codon present in *COX1* transcript (Nadler et al., 2022; Krüger et al., 2023). Additionally, ribosome profiling experiments detected stalling at the AGG codon present in *ND6* mRNA when mtRF1 was deleted in cells (Krüger et al., 2023; Saurer et al., 2023) and *in vitro* mitochondrial translation assays showed release activity of mtRF1 at both AGA and AGG codons (Krüger et al., 2023). This result, however, is not accompanied by a decrease in ND6 synthesis, leaving a question open as to how the recognition of the AGG codon by mtRF1 results in termination (Nadler et al., 2022; Krüger et al., 2023). mtRF1 is also present in species that terminate COX1 and ND6 in conventional STOP codons, suggesting that its function is not limited to the recognition of AGA and AGG as STOP codons. Alternatively, it has been suggested that mtRF1 could recognize the UAG STOP codon placed in the A site after the −1 frameshifting (Nadler and Richter-Dennerlein, 2023).

C12ORF65 and ICT1 lack a codon-recognition domain and likely participate in the ribosome rescue pathway. ICT1 is an integral part of the mitoribosome, but it has been shown to function as a peptidyl hydrolase in its soluble form (Akabane et al., 2014). C12ORF65 can rescue stalled protein synthesis by binding to the A-site on mt-LSU (Desai et al., 2020).

Once the peptide is released, ribosomes are disassembled, and their components become available for a new round of translation. Two ribosome recycling factors have been identified in mitochondria: mtRRF and mtEF-G2, which help split the two mitoribosome subunits (Figure 2C) (Rorbach et al., 2008; Tsuboi et al., 2009; Aibara et al., 2020).

### 3.4 Co-translational insertion of the peptides in the inner mitochondrial membrane

The 13 mtDNA-encoded peptides are core subunits of the OXPHOS complexes, which carry a single or multiple transmembrane domains and are, thus, hydrophobic. The insertion of these proteins in the inner mitochondrial membrane (IMM) occurs co-translationally, mediated by the OXA1 insertase. OXA1 is a member of the Oxa1/YidC/Alb3 protein family, which was originally identified in yeast (Bauer et al., 1994; Hell et al., 2001). Yeast Oxa1 and its human homologue OXA1L are integral proteins in the IMM, which interact with the mitoribosome through their C-terminal domain (Jia et al., 2003; Szyrach et al., 2003). This interaction docks the ribosome to the membrane, facilitating the insertion of the nascent polypeptides in the IMM. In the absence of Oxa1 binding, the peptide exit tunnel of the mitoribosome is blocked by the mitoribosomal subunit mL45 (Itoh et al., 2021), preventing the emergence of the nascent polypeptide. Oxa1 binding to the ribosome displaces mL45 from the exit tunnel (Itoh et al., 2021), allowing the polypeptide to exit only in the proximity of the IMM and minimizing its contact with the mitochondrial matrix.

In yeast, the docking of the mitoribosome to the IMM is further supported by an extension of 21S rRNA (Pfeffer et al., 2015) and two additional IMM receptors: Mba1 (the yeast homologue of mL45) (Preuss et al., 2001; Ott et al., 2006) and Mrx15 (Möller-Hergt et al., 2018), which tether the LSU to the membrane and ensure efficient membrane insertion by the insertase Oxa1 (Möller-Hergt et al., 2018). In human mitochondria, TMEM126A interacts with OXA1L and the mitoribosome, facilitating the insertion of the mitochondrially encoded proteins (Poerschke et al., 2024).

Interestingly, the peptide exit tunnel of the mitoribosome has also been found associated to assembly factors and the protein quality control machinery allowing for an early decision on whether the newly synthesized proteins are assembled into the OXPHOS complexes or are degraded (Singh et al., 2020; Kohler et al., 2023). In this sense, the lack of TMEM126A has been shown to trigger a quality control process in the IMM which degrades the newly synthesized peptides and OXA1L (Poerschke et al., 2024).

## 4 Defects of mitochondrial translation

Mitochondrial translation is crucial to generate the necessary amount of OXPHOS subunits to satisfy the cells’ energy demand. An impairment in mitochondrial protein synthesis, will result in decreased activities of the respiratory chain complexes (complex I-IV) and ATP synthase (Complex V) which are a cause of mitochondrial OXPHOS disorders. The clinical manifestations of these disorders are extremely heterogeneous and can affect any cell type, a single tissue/organ or be multisystemic and can appear at any age. However, the tissues preferentially affected by mitochondrial OXPHOS dysfunctions are those with a higher energy demand, mainly muscle and nervous system, resulting in neurodegenerative diseases, neuromuscular pathologies, metabolic disorders, and aging-related degeneration (Boczonadi and Horvath, 2014; Horvath et al., 2023). Mitochondrial dysfunction is a common hallmark of neurodegenerative diseases. Neurological alterations associated with mtDNA mutations include a very important number of disorders such as mitochondrial encephalomyopathy, chronic progressive external ophthalmoplegia, neurogenic weakness, sensorineural deafness, optic neuropathies or Leigh syndrome.

Mutations in many mitochondrial protein biosynthesis players have been associated with mitochondrial pathologies. The mutations are usually autosomal recessive and can affect mitochondrial rRNA and tRNA encoding genes, tRNA modifying enzymes, mitochondrial aminoacyl-tRNA synthetases, ribosomal protein subunits, ribosome assembly factors and elongation and termination translation factors. In this next section, we will focus on the molecular pathways that lead to a defective mitochondrial protein synthesis and the clinical outcomes caused by these defects.

### 4.1 Mutations in rRNAs

The human mitoribosome includes three RNA molecules, 12S and 16S rRNAs and tRNA^Val^. tRNA^Val^ performs a structural function in the mitoribosomes. The rRNAs, in addition, directly participate in all ribosomal functions including codon recognition, peptidyl transferase activity, and translocation along with the mRNAs (Moran et al., 2023). Defects in the structural components of the mitoribosome, including the rRNA sequences themselves, provoke multiple mitochondrial OXPHOS diseases.

Since all RNA components of the huge ribonucleoparticle that is the mitoribosome, 12S rRNA, 16S rRNA and tRNA^Val^, are encoded in the mtDNA, the clinical manifestations of their defects are subject to the nature of mtDNA genetics: heteroplasmy (the ratio of mutated vs*.* non mutated mtDNA molecules in the cell), threshold effect (level of heteroplasmy at which a phenotypic defect is shown), mitotic segregation (the random reception of mutated and non-mutated mtDNA molecules during cell division), and maternal inheritance (DiMauro and Schon, 2003). In addition, since rRNAs work as general players in mitochondrial translation, their defects should have a general impact on the expression of all mtDNA encoded proteins, affecting, therefore, to complexes I, III, IV and V of the OXPHOS system. Functional defects of the rRNAs provoke primary mitochondrial OXPHOS disorders, usually multisystemic, affecting the cardiac and skeletal muscle, nervous system, liver, kidney, etc. Occasionally, mutations on rRNAs are responsible for secondary mitochondrial diseases being associated with a risk to undergo or an aggravation factor (Ferrari et al., 2021) or even described as a reduced risk factor to suffer a neurological disorder (Hudson et al., 2013).

Although there are dozens of mutations on 12S rRNA associated with a variety of mitochondrial disorders (Table 1), the vast majority are responsible of sensorineural deafness, particularly aminoglycoside-induced and non-syndromic hearing loss (Ferrari et al., 2021). The best characterized are the homoplasmic m.1555A>G and m.1494C>T mutations (Prezant et al., 1993; Zhao et al., 2004), which despite being present in the whole body affect just the hearing apparatus. The penetrance of the m.1555A>G mutation is incomplete, and many patients do not develop hearing loss unless exposed to aminoglycosides. Aminoglycosides, such as kanamycin and streptomycin, are antibacterial agents that inhibit protein synthesis interacting with the A-site of bacterial ribosome. These mutations in 12S rRNA facilitate the interaction of aminoglycoside drugs with the human mitoribosome, disturbing mitochondrial translation (Hamasaki and Rando, 1997). Therefore, exposure to aminoglycosides can induce or aggravate hearing loss in individuals carrying one of those mutations. As a result of the incomplete penetrance, the m.1555A>G mutation would have mostly been phenotypically neutral until the discovery of antibiotics (Pacheu-Grau et al., 2010; 2011) and has become a common mutation in certain populations, especially in Europe where different studies estimate a prevalence of 0,19-0,21% for these pathogenic variants (Bitner-Glindzicz et al., 2009; Himesha et al., 2009; Bellusci et al., 2021).

**TABLE 1:** Representative mutations in mitoribosome RNA components and neurological disorders.

Gene	Mutation	Clinical manifestation
*RNR1*	m.1555A>G	Non-syndromic antibiotic induced hearing loss
	m.1494C>T	Non-syndromic antibiotic induced hearing loss
	m.1095T>C	Aminoglycoside-induced hearing loss*
RNR2	m.2648T>C	Rett Syndrome
	m.2835C>T	Rett Syndrome
	m.3090G>A	Mitochondrial encephalomyopathy
	m.3093C>G	MELAS
	m.3196G>A	AD/PD associated

*RNR1* encodes 12S rRNA; *RNR2* encodes 16S rRNA. AD: Alzheimer’s disease. MELAS: mitochondrial myopathy, encephalopathy, lactic acidosis, and stroke-like episodes. PD: Parkinson’s disease. Rett syndrome: defects in brain development, loss of motor capabilities, CPEO, muscle weakness, and cerebellar dysfunctions. The complete set of rRNA mutations and references can be found in the MITOMAP database (https://www.mitomap.org/MITOMAP).

*m.1095T>C is also a variant of haplogroups M11 and R54.

Additional variants in the *RNR1* gene have been associated to hearing loss or other disorders, although their unequivocal pathogenic role is under discussion, due to their presence in control individuals. In this way, variants such as m.1095T>C, which has also been associated to aminoglycoside-induced hearing loss (Thyagarajan et al., 2000), is a nucleotide change that defines mitochondrial haplogroups M11 and R54 (Ruiz-Pesini and Wallace, 2006), questioning its pathogenic character. Nevertheless, treatment with aminoglycoside antibiotics in cells harboring this variant induced a ten-fold increase in the number of apoptotic cells compared to controls, suggesting its pathogenicity (Muyderman et al., 2012). This situation illustrates a relatively recurrent situation in which the assignment of the pathogenicity to a genetic variant is subject to numerous variables, some of which are inherent to the nature of the mtDNA itself.

On the contrary, only a few disease-causing mutations have been described on the gene *RNR2,* which encodes 16S rRNA (Table 1) (Elson et al., 2015). Mutations in 16S rRNA have been identified as the cause of a serious muscle affectation due to a combined OXPHOS deficiency in skeletal muscle (Elson et al., 2015), a severe hypertrophic cardiomyopathy due to a decreased the stability of the 16S rRNA (Li et al., 2018) or Rett syndrome, a rare pediatric neurological disorder that affects brain development causing progressive loss of motor capabilities, chronic progressive external ophthalmoplegia (CPEO), muscle weakness, and cerebellar dysfunction (Lv et al., 2017), among others. The most representative mutations on 12S rRNA and 16S rRNA molecules associated with neurological disorders are shown on Table 1.

The generation of animal models carrying mutations in mtDNA-encoded genes is limited due to the difficulties in manipulating the mitochondrial genome. These difficulties arise from the characteristics of the organelle and the polyploidy of the mitochondrial genome. Mitochondria are surrounded by a double lipid bilayer membrane, making their “transfection” with exogenous DNA difficult. There are hundreds to thousands of copies of mtDNA in each cell, and a significant number of molecules would need to be targeted to achieve a functional level of heteroplasmy. Finally, recombination of mtDNA is vanishingly rare both in somatic tissues and in the germline (Hagström et al., 2014), complicating the incorporation of exogenous DNA in the mtDNA molecule. A mouse line carrying mutations in 12S rRNA was generated by injecting transmitochondrial embryonic stem cells, carrying the A2379T substitution in 12S rRNA (Marchington et al., 1999). No further studies were published as it did not result in a stable mouse line for analysis (Stewart, 2021).

Finally, although tRNA^Val^ is a structural component of human mitoribosome, it is difficult to associate mutations on tRNA^Val^ to defects on mitoribosomal structure and function since any tRNA dysfunction will necessarily give rise to mitochondrial translation defects. Furthermore, since tRNA^Val^ can be replaced by tRNA^Phe^ as part of the ribosome structure (Rorbach et al., 2016), the human mitoribosome can be built independently of the tRNA^Val^ situation, and mutations in tRNA^Val^ will mostly affect translation elongation.

### 4.2 Mutations in ribosomal subunits

Mutations in the genes encoding mitoribosomal subunits lead to impaired mitochondrial protein synthesis causing combined OXPHOS enzyme deficiencies. Despite the seemingly equivalent function of the mitoribosomal proteins as part of the mitochondrial ribosome and their ubiquitous expression, patients harboring mutations in these proteins present with a wide variety of clinical presentations (detailed in Table 2), which predominantly affect the brain, the heart, and the liver.

**TABLE 2:** Clinical manifestations due to mutations in mitochondrial ribosomal proteins.

Gene	Clinical manifestation	Reference
*MRPS2*	Sensorineural hearing loss, developmental delays, hypoglycemia, lactic acidemia	Gardeitchik et al. (2018)
	Hypoglicemia and lactic acidosis	Liu et al. (2022)
*MRPS7*	Sensorineural deafness, lactic acidemia	Menezes et al. (2015)
*MRPS14*	Increased lactate, Wolff-Parkinson White syndrome	Jackson et al. (2019)
*MRPS16*	Agenesis of corpus callosum and lactic acidosis	Miller et al. (2004)
*MRPS22*	Edema, hypotonia, cardiomyopathy and tubulopathy	Saada et al. (2007)
	Cornelia de Lange-like dysmorphic features, brain abnormalities and hypertrophic cardiomyopathy	Smits et al. (2011b)
	Fatal lactic acidosis, cardiomyopathy and encephalopathy	Baertling et al. (2015)
	Dysmorphism, hypotonia, developmental delay, and LS-like lesions. (Chromosome analyses also showed mosaic down syndrome pattern)	Kılıç et al. (2017)
	Primary ovarian insufficiency	Chen et al. (2018), Jolly et al. (2019)
*MRPS23*	Liver disease	Kohda et al. (2016)
	Delayed growth and development, hearing impairment, hypoglycemia, lactic acidosis, and liver dysfunction	Ittiwut et al. (2023)
*MRPS25*	Mitochondrial encephalomyopathy with syskinetic cerebral palsy and partial agenesis of the corpus callosum	Bugiardini et al. (2019)
*MRPS28*	Intrauterine growth retardation, facial dysmorphism, sensorineural hearing loss and developmental delay	Pulman et al. (2018)
*MRPS34*	Leigh syndrome or Leigh-like syndrome	Lake et al. (2017), Lenzini et al. (2022)
*MRPS39*	Abnormal brain development at birth and infantile-onset Leigh syndrome	Borna et al. (2019), Muñoz-Pujol et al. (2023)
*MRPL3*	Hypertrophic cardiomyopathy, psychomotor retardation	Galmiche et al. (2011)
	Lactic acidosis, sensorineural hearing loss, infantile-onset hypertrophic cardiomyopathy and liver dysfunction	Bursle et al. (2016)
*MRPL12*	Growth retardation and neurological deterioration	Serre et al. (2013)
*MRPL24*	Lactic acidosis, cerebellar atrophy, choreoathetosis of the limbs and face, intellectual disability	Nottia et al. (2020)
*MRPL44*	Infantile onset hypertrophic cardiomyopathy, liver steatosis	Carroll et al. (2013)
	Hypertrophy cardiomyopathy, myopathy, hemiplegic migraine, pigmentary retinopathy, renal insufficiency, and a Leigh-like lesions	Distelmaier et al. (2015)
	Cardiac and skeletal myopathy, neurological involvement	Horga et al. (2021)
	Cardiomyopathy, failure to thrive, hypoglycemia and lactic acidosis	Friederich et al. (2021)
*MRPL50*	Sensorineural hearing loss, chronic kidney disease, left ventricular hypertrophy, Premature ovarian insufficiency	Bakhshalizadeh et al. (2023)

To date, mutations in genes *MRPS2*, *MRPS7*, *MRPS9*, *MRPS14*, *MRPS16*, MRPS22, MRPS23, *MRPS25*, *MRPS28*, *MRPS34* and *MRPS39*, which encode mt-SSU subunits, and genes *MRPL3*, MRPL12, *MRPL24*, *MRPL44* and *MRPL50* which encode mt-LSU subunits have been linked to mitochondrial disorders (reviewed in (Ferrari et al., 2021)). In the majority of these patients, the mutations result in a decrease in the steady-state levels of the affected protein and, as a consequence, the assembly of the ribosome is impaired and results in an overall reduction of protein synthesis. The predominant clinical features include lactic acidosis, sensorineural hearing loss, hypertrophic cardiomyopathy and neurodevelopmental disabilities, nevertheless patients also present specific clinical features such as corpus callosum agenesis, Leigh syndrome, hypoglycemia or ovarian insufficiency.

Given the heterogeneity of phenotypes associated with mutations in mitochondrial ribosomal subunits, the generation and study of animal models is an essential tool to understand how a decrease in mitochondrial translation can result in such an array of clinical features. In this sense, several animal models have been generated to try to reproduce the phenotypes observed in patients and will be discussed below.

Full-body knockout mouse models lacking mitoribosomal subunits are embryonic lethal (Cheong et al., 2020a; 2020b), showing a functional mitochondrial ribosome is essential for development and pointing to the necessity to use different strategies or generate alternative animal models. In this sense, a mouse line carrying a missense mutation in MRPS34, developed cardiac hypertrophy, liver dysfunction, kidney dysfunction and smaller brain (Richman et al., 2015; Lake et al., 2017). The mutation results in decreased levels of MRPS34 and a subsequent reduction in 12S rRNA steady state levels and mt-SSU in heart and liver. Thereafter, mutations in MRPS34 have been identified in patients with Leigh or Leigh-like syndrome (Lake et al., 2017; Lenzini et al., 2022).

Mutations in MRPL3 have been found mutated in four siblings who suffered from hypertrophic cardiomyopathy and psychomotor retardation and subsequently in a patient suffering from lactic acidosis, sensorineural hearing loss, infantile-onset hypertrophic cardiomyopathy and liver dysfunction (Galmiche et al., 2011; Bursle et al., 2016). Interestingly, a spontaneous intronic mutation in the *MRPL3* gene in an inbred mouse colony was found as the cause in an adult-onset neurodegenerative disorder (Cahill et al., 2020), and while the mutation does not reproduce the phenotype observed in patients, this mouse constitutes an excellent model of neurodegenerative disorder due to mutations in the mitochondrial ribosome subunits.

*MRPL24* has been found mutated in a patient suffering from cerebellar atrophy, choreoathetosis of limbs and face, intellectual disability, and Wolff-Parkinson-White syndrome, as a result from a combined CI, CIII and CIV deficiency. The patient was homozygous for a mutation which results in a L91P variant of MRPL24, and almost undetectable levels of the protein in fibroblasts. Reducing the expression levels of *MRPL24* in a zebrafish model (Nottia et al., 2020) leads to impaired ATP production and a decreased basal respiration. Knockdown zebrafish embryos exhibited motor and heart impairment, reproducing the patient’s phenotypes. Interestingly, reintroducing a mutated MRPL24^Leu91Pro^ in the knockdown zebrafish rescues the heart phenotype but not the locomotion suggesting that the mutation impairs mainly motor behavior or a different demand for MRPL24 in different tissues. Likewise, MRPL24 knockdown in *Caenorhabditis elegans* (Ficociello et al., 2023) resulted in reduced locomotion of the worms.

Mutations in *MRPS22* have been associated with a wide variety of clinical symptoms, from hypertrophic cardiomyopathy to edema, encephalopathy or muscle hypotonia (Saada et al., 2007; Smits et al., 2011b; Baertling et al., 2015). In 2018 and 2019, mutations in MRPS22 were identified in patients suffering from primary ovarian insufficiency (Chen et al., 2018; Jolly et al., 2019). To analyze the involvement in of MRPS22 in ovarian physiology, as this phenotype had not been previously associated to mutations in *MRPS22*, its homologue was knocked down in *Drosophila melanogaster* germ cells resulting in agametic ovaries (Chen et al., 2018). Primordial germ cells, which are germ cell precursors, show elevated OXPHOS activity relative to other cell types (Hayashi et al., 2017), possibly explaining their increased sensitivity to mutations in the mitoribosome.

Since then, additional mitoribosomal subunits have been found mutated in patients with ovarian insufficiency. Mutations in *MRPL50* were identified as a cause of a mitochondrial disorder in twin sisters with premature ovarian insufficiency, sensorineural hearing loss and chronic kidney disease (Bakhshalizadeh et al., 2023). The mutation, p.(V112A), destabilizes MRPL50 and as a result patients also showed a global decrease in abundance in proteins of the large ribosomal subunit. Knockdown and knockout of *MRPL50* in *Drosophila melanogaster* recapitulates the patients’ phenotype, as deficiency of the *Drosophila* MRPL50 orthologue leads to stunted ovarian development and small-sized ovaries devoid of germ cells (Bakhshalizadeh et al., 2023). These results support the effect of MRPL50 disruption in the premature ovarian insufficiency suffered by the patients, however, the molecular mechanisms underlying the hearing loss, kidney and heart dysfunction has not directly been validated in this model.

### 4.3 Mutations in tRNAs and tRNA modifying enzymes

tRNA molecules are exceptionally important for decoding the genetic information as they are the tools that *translate* the nucleotide language of mRNAs into the amino acid language of proteins. There is a fabulous machinery in the number of components and complexity to help tRNAs execute that conversion, which is reasonably well conserved throughout evolution among all living organisms. Mitochondrial translation utilizes 22 tRNAs, all of them encoded in mtDNA, to translate the 13 mitochondrial proteins expressed within the organelle (Rackham and Filipovska, 2022).

The synthesis of functional mitochondrial tRNAs requires a complex process and a plethora of different players. In fact, synthesis of all mitochondrial RNAs shares the first steps: transcription on long polycistronic RNAs and processing. After cleavage and release by two devoted endonucleases (RNAse P and ELAC2), mt-tRNAs undergo an extensive list of modifications that give rise to the mature tRNA molecules, ready to be recognized and aminoacylated by their corresponding mitochondrial aminoacyl-tRNA synthetase (ARS2) as a previous and necessary step before entering the mitoribosome (Hallberg and Larsson, 2014). Post-transcriptional modifications are essential for tRNA function to obtain the structural properties to be differentiated by ARS2 with an extremely low possibility of error and to obtain a precise decoding capability (interaction with ribosome, codon-anticodon recognition … ) (Hallberg and Larsson, 2014; Suzuki and Suzuki, 2014). Remarkably, a mt-tRNA molecule just released by action of ELAC2 (3′ end processing) and RNAse P (5’ end processing) is already methylated by action of MRPP1, one of the three subunits of RNAse P. MRPP1 shows a tRNA N1-methyltransferase activity that methylates the ribonucleotide on position nine in mt-tRNAs, forcing a structural change of the tRNA which adopts the cloverleaf formation, crucial for its function (Helm et al., 1999; Hallberg and Larsson, 2014). Thus, mt-tRNA molecules are born modified.

Human tRNAs undergo 18 different modifications affecting 137 positions that are carried out by 34 accessory proteins (Suzuki et al., 2020). The most relevant modifications are taurine modification, thiolation, formylation, pseudouridylation or queuosine modification. For a full list of known mitochondrial tRNA modifications see refs. (Kazuhito and Wei, 2020; Suzuki et al., 2020). An essential modification for the function of tRNAs is the addition of a 3′ CCA extension by the CCA-adding enzyme TRNT1 (Nagaike et al., 2001). This CCA^3’^-OH end will be the acceptor of the amino acid by the action of aminoacyl-tRNA synthetase. In addition, there is a single tRNA modification essential for mitochondrial translation: the synthesis of the initiator tRNA, fMet-tRNA^Met^. Mitochondria contain a unique gene encoding tRNA^Met^, which functions in initiation and elongation. Thus, a portion of Met-tRNA^Met^ is formylated by the mitochondrial methionyl-tRNA formyltransferase (MTFMT) to generate fMet-tRNA^Met^ for initiation (Tucker et al., 2011). The importance of this modification for mitochondrial translation initiation is highlighted by the discovery of mutations in the *MTFMT* gene, which result in Leigh syndrome and a combined OXPHOS deficiency caused by a severe decrease in mitochondrial translation (Tucker et al., 2011; Haack et al., 2014).

There are more than 100 pathogenic mutations on tRNA genes, however, many of those mutations are not at a critical position for tRNA function, and their pathogenicity is a consequence of disturbing the recognition by the mitochondrial tRNA modification machinery (Yarham et al., 2010; Suzuki et al., 2020). In addition, pathogenic mutations in several tRNA modifying enzymes have been identified as a cause of human disorders, illustrating the essential role of mt-tRNA modifications in mitochondrial physiology and pathology. Mutations in mitochondrial tRNA modifying enzymes associated with human disorders and their associated phenotypes have been extensively reviewed in (Chujo and Tomizawa, 2021; Magistrati et al., 2023).

One of these modifying enzymes, mitochondrial translation optimization protein 1 (MTO1), has been identified in patients with cardiomyopathy, lactic acidosis, developmental delay and combined or isolated respiratory chain deficiency (Ghezzi et al., 2012; Baruffini et al., 2013; Charif et al., 2015; Martín et al., 2017; Kamps et al., 2018; O’Byrne et al., 2018; Li et al., 2019; Seo et al., 2019; Luo et al., 2021; Zhou et al., 2022; Monda et al., 2023). MTO1 synthesizes the taurinomethyl (τm5U) modification of the anticodon of mitochondrial tRNA^Leu^, tRNA^Trp^, tRNA^Lys^, tRNA^Gln^ and tRNA^Glu^, which is essential for the accurate decoding of their corresponding codons (Suzuki et al., 2002; Suzuki and Suzuki, 2014). Several animal models carrying deletions of the homologues of *MTO1* in zebrafish (Zhang et al., 2021), mice (Becker et al., 2014) or the worm *Caenorhabditis elegans* (Navarro-González et al., 2017) have been generated to understand the molecular mechanisms underlying MTO1 defects. Zebrafish and *C. elegans* present low levels of the MTO1 target tRNAs which resulted in a reduced mitochondrial translation. As a result of these defects, mouse and zebrafish *MTO1* knock-out animals develop a cardiac defect, reproducing the clinical phenotype observed in patients.

About half of the mtDNA mutations causing mitochondrial OXPHOS diseases in humans occur in tRNA genes and show an extraordinary range of clinical presentations. This variability can be explained, at least in part, by different percentage of heteroplasmy (Boczonadi and Horvath, 2014). However, heteroplasmy alone cannot explain the variability and additional factors must be involved in the modulation of the expression of particular point mutations on tRNA molecules. A paradigmatic case comes from the 3243G>A mutation in mitochondrial tRNA^Leu(UUR)^. This mutation has been associated to MELAS (Mitochondrial Encephalopathy Lactic Acidosis and Stroke-like episodes) (Goto et al., 1990), deafness, and diabetes (Ouweland et al., 1992) or progressive external ophthalmoplegia (Moraes et al., 1993).

As mentioned above, the difficulties in the genetic manipulation of mtDNA have hindered the generation of animal models carrying mutations in mitochondrially encoded genes. A mouse model with pathogenic mutations in tRNA^Lys^ was generated by fusing pronucleus-stage embryos with enucleated cytoplasts carrying a G7731A mutation in the tRNA (Shimizu et al., 2014). This mouse allowed the authors to analyze the transmission pattern of the mutation in the offspring and the heteroplasmy threshold for the mutation to have a detrimental effect on mitochondrial function. A second mouse line carrying an heteroplasmic pathogenic mutation in tRNA^Ala^ was established through breeding mice that carry a mitochondrial DNA polymerase (POLG) without proofreading activity, and selecting for mutant lineages (Kauppila et al., 2016). This tRNA^Ala^ mutant mouse line has allowed to analyze the transmission and threshold of the mutation in different tissues, as well as genetic strategies to improve the phenotype of the mice (Kauppila et al., 2016; Filograna et al., 2019).

The most representative mutations affecting mitochondrial tRNA function and tRNA modifying enzymes and that are associated with neurological disorders are shown on Table 3.

**TABLE 3:** Representative mutations affecting mitochondrial tRNA function and mitochondrial tRNA modifying enzymes that result in neurological disorders.

*tRNA-modifying enzymes*		
Gene	Modifying activity	Main clinical manifestation
*PUS1*	Pseudouridylation	Myopathy, Lactic Acidosis, Sideroblastic Anaemia (MLASA)
*TRMU*	Thiolation	Reversible infantile liver failure
*MTO1*	Taurinonethylation	Lactic acidosis, Hypertrophic cardiomyopathy
*MTFMT*	Methionyl-tRNAMet formyltransferase	Leigh syndrome
*TRNT1*	CCA addition	Lactic acidosis, sideroblastic anaemia, developmental delay, deafness, microcephaly with cortical atrophy; Ataxia, hypotonia, ptosis, ophthalmoplegia
*TRIT1*	Isopenthynilation	Encephalopathy and myoclonic epilepsy
*TRMT5*	Methylation	Lactic acidosis, hypotonia, falilure to thrive and hypertrophic cardiomyopathy; Lactic acidosis, exercise intolerance, weakness, peripheral neuropathy
*GTPBP3*	Taurinomethylation	Cardiomyopathy, lactic acidosis

tRNA mutations		
Gene	Mutation	Clinical manifestation
*MTTF*	m.583G>A	MELAS
	m.616T>C	Maternally inherited epilepsy
*MTTV*	m.1606G>A	Ataxia, Myoclonus and Deafness
	m.1644G>A	Leigh Syndrome/MELAS
*MTTL1*	m.3243A>G	MELAS/Leigh Syndrome/deafness/MIDD/SNHL/CPEO/FSGS/multi-organ dysfunction
	m.3271T>C	MELAS/DM/MERRF-like
*MTTI*	m.4308G>A	CPEO
*MTTQ*	m.4332G>A	Encephalopathy/MELAS
*MTTM*	m.4450G>A	Myopathy/MELAS/Leigh Syndrome/EXIT
*MTTW*	m.A5537insT	Leigh Syndrome
*MTTN*	m.5690A>G	CPEO, ptosis, proximal myopathy
*MTTS1*	m.7471C>CC	PEM/AMDF/Motor neuron disease-like
	m.7510T>C	SNHL
*MTTK*	m.8344A>G	MERRF/depressive mood disorder/leukoencephalopathy
*MTTG*	m.10010T>C	PEM
*MTTH*	m.12147G>A	MERRF-MELAS/Encephalopathy
*MTTL2*	m.12294G>A	CPEO/EXIT, Ophthalmoplegia
*MTTD*	m.14709T>C	MM + DMDF/Encephalomyopathy/Dementia, diabetes, ophthalmoplegia
*MTTT*	m.15923A>G	LIMM/MERRF

AMDF: ataxia, Myoclonus and Deafness; CPEO: chronic progressive external ophthalmoplegia; DM: diabetes mellitus; DMDF: Diabetes Mellitus and DeaFness; EXIT: exercise intolerance; FSGS: focal segmental glomerulosclerosis; LIMM: lethal infantile mitochondrial myopathy; MELAS: mitochondrial myopathy, encephalopathy, lactic acidosis, and stroke-like episodes; MERRF: myoclonic epilepsy and ragged red muscle fibers; MIDD: maternally inherited diabetes and deafness; MM: mitochondrial myopathy; PEM: progressive encephalopathy; SNHL: Sensorineural Hearing Loss. The complete set of mutations affecting mitochondrial tRNAs and references can be found in the MITOMAP database (https://www.mitomap.org/MITOMAP).

### 4.4 Mutations in aminoacyl-tRNA synthetases

Mitochondrial aminoacyl-tRNA synthetases (aaRSs) are the last enzymes acting on tRNAs in order to obtain their final maturation state and functional adequacy before their binding to mtEF-Tu and entry to the A-site in the mitoribosomes. Mitochondrial aaRSs, named ARS2, catalyze the binding of amino acids to their cognate tRNA and constitute one of the two supporting pillars of protein synthesis fidelity. As occurs with cytosolic aminoacyl-tRNA synthetases (ARS1), tRNA aminoacylation by ARS2 requires ATP to first generate an AMP-amino acid derivative, and then binding the amino acid to the cognate tRNA, releasing AMP. aaRSs must possess a strict discriminating ability to recognize the correct amino acid and its corresponding tRNA among a pool of very similar tRNA molecules, to guarantee translation accuracy (Turvey et al., 2022).

In humans, there are two groups of nuclear genes that code for either the cytosolic aaRSs or mitochondrial aaRSs with just two exceptions. The mitochondrial and cytosolic versions of GlyRS and LysRS are encoded by a single gene that expresses two versions of their respective enzymes, with and without mitochondrial targeting sequence. This is achieved by using two different translation initiation sites in the case of GlyRS and by alternative mRNA splicing in the case of LysRS (Sissler et al., 2017).

One amino acid–one aminoacyl tRNA synthetase is almost a dogma in translation. In the cytosol, the 20 cellular proteinogenic amino acids require 20 cytosolic aaRSs (Sissler et al., 2017). In human mitochondria, however, there are only 19 aaRSs, lacking the glutaminyl-tRNA synthetase (QARS2). The biosynthesis of mitochondrial Gln-tRNA^Gln^ follows an indirect pathway in which the tRNA^Gln^ is first misaminoacylated by a non-discriminant EARS2 to Glu-tRNA^Gln^, followed by a transamidation reaction to Gln-tRNA^Gln^, using glutamine as amide donor. This transamidation reaction is catalyzed by the heterotrimeric complex glutamyl-tRNA^Gln^ amidotransferase, hGatCAB (Nagao et al., 2009).

Since the identification of the first pathogenic mutation in DARS2 in 2007 (Scheper et al., 2007), defects in mitochondrial aminoacyl-tRNA synthetases have emerged as prevalent cause of human diseases. Mutations in all ARS2 genes have been linked to human diseases (collected in misynpat.org (Moulinier et al., 2017)). They all are recessive mutations and lead mainly to neurological disorders, although with pleiotropic effects. Interestingly, and perhaps expected, the diversity of pathologies is much wider for mutations in mt-tRNAs than in ARS2, probably because of the much higher number of possibilities of functional alterations within tRNA molecules compared to aaRSs. Dysfunction of eight ARS2 leads to encephalopathies, defects in another four ARS2 cause leukodystrophies, two are responsible for causing Perrault syndrome (sensorineural hearing loss in males and females and ovarian dysfunction in females) and mutations in additional single aaRSs can also result in hearing loss or deafness and intellectual disability, all affecting the central nervous system (Sissler et al., 2017). Mitochondrial aaRS have also been associated with disorders that do not have neurological manifestations such as cardiomyopathies, the MLASA (Mitochondrial Myopathy, Lactic Acidosis, and Sideroblastic Anemia) (Shahni et al., 2013) or HUPRA (hyperuricemia, pulmonary hypertension, renal failure, and alkalosis) syndromes (Belostotsky et al., 2011; Rivera et al., 2013).

A particular case of defects in the aminoacylation of mitochondrial tRNAs is derived from mutations in the heterotrimeric amidotransferase complex GatCAB, responsible of the synthesis of the Gln-tRNA^Gln^. Although this complex is not a *bona fide* mitochondrial aminoacyl tRNA synthetase, its shares the function with aminoacyl-tRNA synthetases. There have been pathogenic mutations identified in all three subunits of the GatCAB complex, QRSL1, GatB and GatC (Kohda et al., 2016; Friederich et al., 2018; Kamps et al., 2018), but none of them, however, result in a neurological affection despite having an aminoacylation defect of a mitochondrial tRNA. Patients presented with lactic acidosis and a metabolic cardiomyopathy and died shortly after birth. All patients showed a reduced mitochondrial translation due to a defective Gln-mt-tRNA^Gln^ acylation (Friederich et al., 2018).

Different groups have generated animal models to study the molecular mechanisms behind the pathologies due to mitochondrial aaRSs. *Drosophila melanogaster* WARS2 knock-down displays aminoacylation defects similar to those observed in patients (Maffezzini et al., 2019). *dFARS2* knock down in *Drosophila* leads to a developmental delay, an abnormal brain morphology and induces seizures (Fan et al., 2021), showing FARS2 function is crucial for the maintenance of neuronal function and reproducing the phenotypes observed in patients. MARS2 mutant flies display a mild reduction in complex I activity with a concomitant production of reactive oxygen species (ROS) (Bayat et al., 2012). Interestingly, the brain tissue of these flies accumulates lipid droplets, suggesting a defect in lipid metabolism that has also been observed in patients carrying mutations in tRNA^Met^ and COX3 (Bortot et al., 2009). Similarly, *SARS2* knock down in *Drosophila* affects its viability, longevity, motility and tissue development, and induces lactic acidosis and ROS accumulation (Guitart et al., 2013). These models not only show *Drosophila* is an excellent model to study the pathophysiology of these disorders, as they reproduce the defects observed in patients, but can also be used to test potential therapies. In this way, it was shown that the phenotypes for both MARS2 and SARS2 mutant flies can be partially reversed by the administration of antioxidants (Bayat et al., 2012; Guitart et al., 2013).

Zebrafish, *Danio rerio*, have also been used as models of reduced mitochondrial tRNA aminoacylation. Several groups have knocked down the expression of *FARS2* (Li et al., 2021; Chen et al., 2022), *RARS2*, *VARS2* (Kayvanpour et al., 2022), *WARS2* (Wang et al., 2016) and *YARS2* (Jin et al., 2021) in zebrafish. A decrease in FARS2, RARS2, VARS2 or YARS2 results in neurological alterations in zebrafish, reproducing the central nervous system phenotypes observed in patients. These zebrafish models also show different degrees of muscle and heart involvement, and vascular development problems.

Full body knock-out mouse models of aaRSs are embryonic lethal, and the mouse models that have been studied to date have depleted the expression of aaRSs in specific tissues. Conditional DARS2 knockout mice in heart and muscle developed cardiac hypertrophy and died prematurely (Dogan et al., 2014). To study the effect of DARS2 depletion in the central nervous system, in a second work by the same group, DARS2 expression was knocked out in forebrain-hippocampal neurons or myelin-producing cells (Aradjanski et al., 2017). Loss of DARS2 in adult neurons lead to a strong mitochondrial dysfunction accompanied by an early inflammation response and progressive loss of cells. Defects that were not observed when DARS2 was knocked out in myelin-producing cells. To model the hearing loss in patients with Perrault syndrome, HARS2 was knocked-out in mouse cochlear hair cells (Xu et al., 2021). HARS2 knock-out led to progressive hearing loss due to hair cell synaptopathy and apoptosis, triggered by the mitochondrial damage and elevated ROS production. Due to the lethality of the full-body aaRSs knock-outs and the necessity to develop tissue specific models, these knock-out mice can’t be used to study the pleiotropic effects of a given mutation in different tissues. Agnew et al. identified and characterized a mouse model harboring a hypomorphic mutation in the *WARS2* gene (Agnew et al., 2018). These mice develop progressive tissue-specific pathologies, including hearing loss, adipose tissue dysfunction, and hypertrophic cardiomyopathy. The advances in CRISPR/Cas gene editing should allow us to generate mice harboring patient specific variants, which will be a more accurate model for the study of these disorders.

### 4.5 Mutations in translation factors

Mitochondrial translation factors play an essential role in the biogenesis of the OXPHOS complexes by regulating and facilitating protein synthesis in the mitoribosome. Mutations in translation elongation factors mtEF-Tu, mtEF-Ts, mtEF-G1 and GUF1 have been identified as a cause of mitochondrial disorders in patients, which in general present neurological symptoms as happens with the patients with mutations in aaRSs.

mtEF-Tu, encoded by the gene *TUFM*, is a highly conserved protein that uses the energy from GTP hydrolysis to deliver the aminoacyl-tRNAs (aa-tRNA) to the A site of the mitochondrial ribosome during the elongation phase of translation. The crystallographic structure of mtEF-Tu shows the presence of three domains: I is a Mg^2+^ GTPase domain, II is an aa-tRNA binding domain and III, a mtEF-Ts binding domain (Andersen et al., 2000).

Valente et al., reported on a baby affected by severe lactic acidosis and rapidly progressive, fatal encephalopathy with severe infantile macrocystic leukodystrophy with micropolygyria caused by a R339Q amino acid variation in mtEF-Tu (Valente et al., 2007). A similar clinical presentation was described by Di Nottia et al., who reported on a baby with severe infantile macrocystic leukodystrophy with micropolygyria caused by a homozygous c.964G>A (p.G322R) mutation in *TUFM* (Nottia et al., 2017). Both mutations result in a severely decreased mitochondrial protein synthesis and as a result, these patients presented a combined defect in the activity of mitochondrial complexes I and IV (Valente et al., 2007; Nottia et al., 2017). Both mutations are located on domain II of mtEF-Tu. Further *in vitro* analysis demonstrated that R336Q prevents proper binding to the aa-tRNAs and, thus, formation of the ternary complex GTP:mtEF-Tu:aa-tRNA (Valente et al., 2009; Akama et al., 2010).

Hershkovitz et al. described a new homozygous missense variant, H115P, in domain I of the mtEF-Tu protein (Hershkovitz et al., 2019). Bioenergetic analysis of the muscle biopsy revealed a combined mitochondrial defect in the activity of complexes I, I + III and IV. Interestingly, this patient exhibited lactic acidosis and a dilated cardiomyopathy without neurological symptoms, which were presented by the previous patients. The authors proposed that the distinct clinical presentation resulted from the mutation’s location on domain I, rather than domain II, likely impacting the stability of the mtEF-Tu:mtEF-Ts complex and GDP-GTP exchange (Hershkovitz et al., 2019). Additional mutations in the mtEF-Tu have been described, a homozygous mutation L147H and a compound L147H/Y54X, in patients with childhood-onset mitochondrial respiratory chain complex deficiencies (Kohda et al., 2016). Patients presented with lactic acidosis, hyperammonemia and abnormalities of the basal ganglia on brain MRI or intrauterine growth retardation, premature birth, respiratory failure, hypotonia and lactic acidosis.

mtEF-Ts, encoded by gene *TSFM*, is a guanine exchange factor responsible for the regeneration of mtEF-Tu:GTP from the inactive mtEF-Tu:GDP. A publication from the group of Eric Shoubridge reported two unrelated patients with a homozygous missense substitution in *TSFM*, R333W (Smeitink et al., 2006). This amino acid is located in the C-terminal domain, in an evolutionarily conserved site essential for the interaction with mtEF-Tu and, interestingly, both mtEF-Ts and mtEF-Tu protein levels were decreased in patients’ fibroblasts. Bioenergetic analysis revealed a complex I, III and IV defect caused by a decreased in mitochondrial protein synthesis. Interestingly, the synthesis of the different mtDNA-encoded polypeptides is affected to varying degrees as a result of the *TSFM* mutation. Despite carrying the same homozygous mutation, the clinical presentation of both patients was remarkably different (Smeitink et al., 2006), suggesting the presence of potential genetic modifiers and illustrating the variability observed in mitochondrial disease patients. Patient one presented an encephalomyopathy, muscle weakness, hypotonia, rhabdomyolysis, and epilepsy. Patient two presented hypertrophic cardiomyopathy, but the neurological examination and brain imaging were normal.

The clinical spectrum associated to mutations in mtEF-Ts was expanded by additional studies, which reported patients with intrauterine growth retardation, neonatal lactic acidosis, liver dysfunction and multiple respiratory chain deficiency in muscle (Vedrenne et al., 2012), patients with infantile-onset mitochondrial cardiomyopathy, progressing to juvenile-onset Leigh syndrome, neuropathy, and optic atrophy or optic atrophy and a loss of myelinated axons (Ahola et al., 2014), patients with ataxia and non-obstructive cardiomyopathy (Emperador et al., 2017) or encephalocardiomyopathy and sensorineural hearing loss (Scala et al., 2019), among others.

mtEF-G1, encoded by gene *GFM1*, is the third elongation factor necessary for mitochondrial translation. mtEF-G1 plays an essential role in facilitating the translocation of peptidyl-tRNA from the P-site to the A-site of the ribosome, thereby vacating the A-site for the incorporation of a new aa-tRNA. Coenen *et al.* were the first to describe a family with mutations in mtEF-G1 leading to disease (Coenen et al., 2004). This group identified two patients with a mutation in a conserved amino acid position in the GTP binding domain of mtEF-G1. Both patients died early after birth and displayed similar clinical presentations marked by lactic acidosis, severe liver dysfunction and altered brain morphology. Patient fibroblasts exhibited a significant reduction in mitochondrial protein synthesis accompanied by an impairment in the assembly of mitochondrial complexes I, III, IV, and V. A similar clinical presentation was observed by Antonicka *et al.* in two siblings with compound heterozygous mutations in the mtEF-G1 protein, S321P and L607X, with growth retardation, lactic acidosis and liver dysfunction, (Antonicka et al., 2006). The authors suggested the S321P substitution, between domains one and two of the protein could affect nucleotide binding or hydrolysis while the second one truncates the protein.

Smits et al. reported an additional patient carrying a homozygous R250W mutation in mtEF-G1, which is presumed to hamper ribosome-dependent GTP hydrolysis (Smits et al., 2011a). Similar to the previous cases, the patient died at an early age. This patient, however, had a slightly different clinical presentation and did not show significant hepatic or muscular involvement. Instead, he presented with encephalopathy, which was followed by rapid neurological degeneration and epilepsy. Although bioenergetic analysis of the muscle biopsy did not reveal a decrease in OXPHOS activities, a clear mitochondrial defect was evident when the patient’s fibroblasts were used to measure mitochondrial complex activity. The patient exhibited a combined complexes I, III, and IV defect caused by a decreased mitochondrial protein synthesis in fibroblasts (Smits et al., 2011a). The clinical phenotype of this patient is shared by patients that carry mutations R47X/M496R (Valente et al., 2007), which were characterized by neurological failure and lactic acidosis.

Mitochondrial translation was similarly decreased in all investigated patients with mutations in mtEF-G1. Surprisingly, this decrease was not uniform for all OXPHOS subunits, as was found in patients carrying mtEF-Tu mutations. In general, mtEF-G1 patients showed an overall decrease in the rate of mitochondrial translation, with the expression of subunits ND5, ND6, COX1, COX2 and COX3 generally being the lowest. ND3 expression, on the contrary, was often increased (Smits et al., 2011a). A common feature of the disease caused by mutations in mtEF-G1 is the muscular symptoms are relatively mild in all cases, there is however, a wide variation in the clinical symptoms due to mutations in mtEF-G1, with patients having mainly a hepatic presentation or others presenting with neurological symptoms.

In an effort to understand the phenotype-genotype correlations in these patients, the mutations have been modelled on the crystal structure of mtEF-G1 (Galmiche et al., 2012). Hepatic failure was associated with mutations located in the central part of the protein while mutations associated with encephalopathy were located in peripheral regions of the protein. This prediction suggests affecting different functional domains of mtEF-G1 has tissue-specific consequences, despite the general function of mtEF-G1 in mitochondrial translation, and points to compensatory or regulatory mechanisms as responsible for the differences in clinical presentations.

Since then, other groups have reported numerous patients with mutations in *GFM1* (Balasubramaniam et al., 2011; Calvo et al., 2012; Simon et al., 2017; Bravo-Alonso et al., 2019; Barcia et al., 2020; Su and Wang, 2020; You et al., 2020; Khan et al., 2022; Aleksic et al., 2024).

*Gfm1* knock-in and knock-out mouse models were developed to study the underlying molecular mechanisms of the disease and to test potential therapies (Molina-Berenguer et al., 2022). Knock-in mice harboured the missense mutation R671C, previously identified in patients with encephalopathy. This amino acid change in mice causes a mild complex IV deficiency in the liver associated with a reduced mitochondrial translation rate. Mice did not show motor dysfunction and had normal OXPHOS activities in brain, skeletal muscle and heart and did not reproduce the encephalopathy observed in the patients. Crossing the R671C mice to mice carrying a knocked-out *Gfm1* allele, resulted in more severe phenotypes that better recapitulated what was observed in patients. R671C/- mice showed a more pronounced CI and CIV deficiency, both in liver and brain and will be a useful model to further investigate the pathophysiological mechanisms behind the mutations in mtEF-G1.

mtEF4/GUF1, encoded by the gene *GUF1*, is an evolutionarily conserved mitochondrial GTPase that controls the fidelity of translation under stress situations. A work published by Alfaiz *et al.* identified mutations in GUF1 in siblings from a consanguineous family affected by West syndrome, which is characterized by infantile spasms, pathognomonic hypsarrhythmia and developmental regression (Alfaiz et al., 2016).

In addition to the general translation factors, certain transcripts require the assistance of gene-specific translational activators for their translation in the mitoribosome. This is the case of *COX1* mRNA and its translational activator TACO1 (Translational Activator of COX1), which is the only translational activator identified in mammalian mitochondria to date. TACO1 was first identified in patients suffering late-onset Leigh syndrome and a complex IV deficiency (Weraarpachai et al., 2009). The patients carry a homozygous one-base-pair insertion in *TACO1* that results in a frameshift and the creation of a premature stop codon. The complex IV deficiency in the patients is due to a specific defect in the synthesis of COX1, which results in very low levels of fully assembled complex. A second report associated mutations in TACO1 in two additional families with late-onset Leigh syndrome (Oktay et al., 2020). A third report described the presence of TACO1 mutations as the cause of an adult-onset slowly progressive spastic paraparesis with cognitive impairment and leukoencephalopathy, expanding the clinical phenotypes associated to mutations in this gene (Sferruzza et al., 2021).

Mice carrying a homozygous point mutation in the *Taco1* gene have an isolated complex IV deficiency and develop a late-onset syndrome with visual impairment, motor dysfunction and cardiac hypertrophy (Richman et al., 2016). These mice recapitulate the defects observed in patients and, thus, provide a useful model for the study of the molecular basis of the tissue-specific defects observed in patients and the development of potential therapies.

Release factor C12ORF65 has also been identified as a cause of mitochondrial disease in over 25 patients. C12ORF65 belongs to the family of mitochondrial class I peptide release factors together with mtRF1a, mtRF1 and ICT1. C12ORF65 does not exhibit peptidyl-tRNA hydrolase activity, but most likely plays role in recycling abortive peptidyl–tRNA species. The first patients with pathogenic mutations in C12ORF65 were reported by Antonicka *et al.* (Antonicka et al., 2010)*.* Both presented with Leigh syndrome, optic atrophy and ophthalmoplegia due to a deletion causing the appearance of a premature stop codon in *C12ORF65*. These patients show a general decrease in mitochondrial protein synthesis and a strong mitochondrial complex I, IV and V assembly defect. Since the identification of the first patients, several groups have reported additional pathogenic mutations in C12ORF65 (Shimazaki et al., 2012; Buchert et al., 2013; Heidary et al., 2014; Pyle et al., 2014; Spiegel et al., 2014; Tucci et al., 2014; Wesolowska et al., 2015; Imagawa et al., 2016; Nishihara et al., 2017). The clinical presentation of these patients varies, however, optic atrophy, peripheral neuropathy, and spastic paraparesis are common findings to most patients.

### 4.6 Mutations in ribosome assembly factors

A decreased mitochondrial translation and OXPHOS deficiency can also stem from defects in ribosome assembly. To date mutations in ERAL1, the protease CLPP, the methyltransferase MRM2 and the helicase DHX30 have been associated with human disorders.

*ERAL1* is the gene that encodes the Era-Like 12S rRNA chaperone 1 or ERAL1. ERAL1 is essential for the assembly of the mt-SSU (Dennerlein et al., 2010; Uchiumi et al., 2010). A missense mutation in *ERAL1* was identified in three unrelated women from a small village in the Netherlands with Perrault syndrome. All patients presented sensorineural hearing loss in addition to fertility disorders, such as premature menopause or primary amenorrhea (Chatzispyrou et al., 2017). As a consequence of this mutation patients’ fibroblasts show a defective assembly of the small mitoribosomal subunit, reduced 12S rRNA levels and a compromised mitochondrial function. To demonstrate the role of ERAL1 in fertility, the authors knocked-down the *ERAL1* homologue in *C. elegans.* Knock-down of worm *ERAL1* resulted in a decreased mitochondrial respiration and an impaired egg production, confirming the essential role of ERAL1 in mitochondrial function and fertility (Chatzispyrou et al., 2017).

Interestingly, mutations in the mitochondrial protease CLPP have been identified as a cause of Perrault syndrome as well (Jenkinson et al., 2013; Demain et al., 2017; Faridi et al., 2024). The work on *Clpp* knock-out mice has shown this protease has an essential role in mitochondrial protein synthesis by regulating the levels of ERAL1 (Szczepanowska et al., 2016). *Clpp* knock-out mice accumulate ERAL1, which remains bound to the small ribosomal subunit preventing mt-SSU maturation and its assembly into a functional mitoribosome. Moreover, the *Clpp* deletion in mice represents a faithful model of Perrault syndrome, displaying infertility due to a follicular and spermatic differentiation failure (Gispert et al., 2013).

MRM2, is a uridine 2′-O-methyltransferase for the U1369 position of the mitochondrial 16S rRNA in humans (Rorbach et al., 2014). Methylation by MRM2 is essential in the late stages of mt-LSU biogenesis. In its absence, 16S rRNA is unstructured and the mt-LSU accumulates in immature assembly states (Rebelo-Guiomar et al., 2022). A patient with a homozygous G189R substitution in *MRM2* developed a MELAS-like syndrome manifesting with childhood-onset progressive encephalomyopathy and stroke-like episodes (Garone et al., 2017). *MRM2* has also been found mutated in two families with progressive dystonic features and a neurodevelopmental disorder with involuntary movements (Shafique et al., 2023). To model the consequences of a decreased 16 rRNA methylation, *DmMRM2* was knocked down in *Drosophila melanogaster*. Downregulation of *DmMRM2* led to a developmental delay and lethality during the pupal stage due to decreased OXPHOS subunits (Rebelo-Guiomar et al., 2022).

Over 40 patients have been identified with mutations in the gene that encodes the helicase DHX30. Patients are affected by global developmental delay, intellectual disability, severe speech impairment and gait abnormalities (Lessel et al., 2017; Mannucci et al., 2021). To model the disorder, Mannucci et al. generated *dhx30* KO zebrafish, which had a social behavioral deficit with altered sleep-wake activity, which is consistent with the neurodevelopmental disorder in DHX30 patients. The precise function of DHX30 on mitoribosome assembly remains to be identified.

### 4.7 Mutations that affect ribosome tethering to the membrane and the coordination of translation and OXPHOS assembly

As explained in section 4.4, the newly synthesized peptides are inserted co-translationally to the membrane. This insertion is mediated by OXA1L, which tethers mitochondrial ribosomes to the IMM and assists in the insertion of the polypeptides into the membrane (Hell et al., 2001; Jia et al., 2003; Szyrach et al., 2003). Mutations in OXA1L have been identified by whole exome sequencing in patients suffering from a severe childhood-onset encephalopathy (Thompson et al., 2018). Mitochondrial protein synthesis is not affected in patient fibroblasts, but the stability of all mitochondrially encoded proteins is decreased, which is consistent with the role of OXA1L in the insertion of the polypeptides in the IMM. Interestingly, and despite the general role of OXA1L in ribosome tethering to the membrane, the OXPHOS activities are not equally decreased in all patient tissues, showing an isolated complex I deficiency in the nervous system and a combined defect of complexes I, IV and V in skeletal muscle (Thompson et al., 2018). These results suggest that the insertase machinery may vary between tissues and that further research is needed to identify its components.

Mutations in factors that coordinate COX1 translation with its assembly into complex IV have also been described as the cause of human disorders. hCOA3 (MITRAC12, CCDC56), the homologue of yeast Cox25/Coa3 (Szklarczyk et al., 2012), is a transmembrane protein in the IMM that interacts with newly synthesized COX1 and the mitoribosome, and participates in the initial steps of complex IV assembly (Mick et al., 2012; Clemente et al., 2013; Busch et al., 2019). COA3 forms a complex that has been termed MITRAC (mitochondrial translation regulation assembly intermediate of cytochrome *c* oxidase) which links the initial steps in complex IV assembly with COX1 translational regulation. These MITRAC complexes additionally include C12orf62 (Mick et al., 2012), the homologue of yeast Cox14 (Szklarczyk et al., 2012). Mutations in both *COA3* and *C12orf62* result in a reduced translation of COX1, and have been associated to neuropathy, exercise intolerance, obesity, and short stature or neonatal lactic acidosis, respectively (Weraarpachai et al., 2012; Ostergaard et al., 2015). The feedback loop that coordinates Cox1 synthesis and complex IV assembly has been extensively characterized in *Saccharomyces cerevisiae* models (Timón-Gómez et al., 2018). Additionally a *D. melanogaster* model of COA3 deficiency reproduced the isolated complex IV defect caused by mutations in hCOA3 (Peralta et al., 2012).

## 5 Conclusion and future prospects

During the past 2 decades our understanding of the mitochondrial ribosome structure, the process and regulation of mitochondrial translation and the assembly of the mitochondrial ribosomes has greatly advanced and recent reports have revealed the mechanisms for the co-translational insertion of the proteins into the inner mitochondrial membrane and the quality control mechanisms that take place during this insertion process. In addition, next-generation techniques have improved the diagnosis and identification of mutations in patients with mitochondrial disorders due to a deficient protein synthesis, which has allowed for the identification of genes also involved in the process. These disorders are multisystemic, many of them with neurological symptoms, and include Leigh syndrome, hearing loss or hypertrophic cardiomyopathy among others.

Despite the advances, our knowledge of the translation process and the factors involved in it is still limited, and, in particular, our knowledge concerning the molecular mechanisms behind the pathologies is far for complete. The variability in clinical outcomes due to defects in mitochondrial protein synthesis is remarkable, despite the common underlying defect in mitochondrial translation and OXPHOS activities. Animal models have proven a valuable tool in understanding the molecular mechanisms behind many human disorders, and they allow for the study of tissue-specific consequences of a given gene defect. This is especially valuable in the disorders, such as those due to a defective protein synthesis, were the affected organs and the severity of the symptoms are very variable, even in patients carrying mutations in the same gene. In the future, the use of gene editing techniques to generate animal models carrying patient mutations will be instrumental in comprehending the molecular mechanisms behind the disorder and the different consequences of a mutation in each tissue of the organism. To date, there is no cure for mitochondrial disorders and the generation and study of animal models will additionally allow to identify targets and test potential therapies that could improve the patients’ symptoms.
